# Collateral Glucose-Utlizing Pathwaya in Diabetic Polyneuropathy

**DOI:** 10.3390/ijms22010094

**Published:** 2020-12-24

**Authors:** Hiroki Mizukami, Sho Osonoi

**Affiliations:** 1 Department Pathology and Molecular Medicine, Hirosaki University Graduate School of Medicine, 5 Zaifu-cho, Hirosaki, Aomori 036-8562, Japan; s.osonoi@hirosaki-u.ac.jp; 2 Department of Endocrinology and Metabolism, Hirosaki University Graduate School of Medicine, 5 Zaifu-cho, Hirosaki, Aomori 036-8562, Japan

**Keywords:** diabetic polyneuropathy, glycolysis, oxidative stress, polyol pathway, hexosamine biosynthetic pathway, advanced glycation end-products, PKC, glucosamine, pentose phosphate pathway, anaerobic glycolytic pathway

## Abstract

Diabetic polyneuropathy (DPN) is the most common neuropathy manifested in diabetes. Symptoms include allodynia, pain, paralysis, and ulcer formation. There is currently no established radical treatment, although new mechanisms of DPN are being vigorously explored. A pathophysiological feature of DPN is abnormal glucose metabolism induced by chronic hyperglycemia in the peripheral nerves. Particularly, activation of collateral glucose-utilizing pathways such as the polyol pathway, protein kinase C, advanced glycation end-product formation, hexosamine biosynthetic pathway, pentose phosphate pathway, and anaerobic glycolytic pathway are reported to contribute to the onset and progression of DPN. Inhibitors of aldose reductase, a rate-limiting enzyme involved in the polyol pathway, are the only compounds clinically permitted for DPN treatment in Japan, although their efficacies are limited. This may indicate that multiple pathways can contribute to the pathophysiology of DPN. Comprehensive metabolic analysis may help to elucidate global changes in the collateral glucose-utilizing pathways during the development of DPN, and highlight therapeutic targets in these pathways.

## 1. Introduction

Diabetes leads to various neuropathies, such as diabetic polyneuropathy (DPN) [1,2]. DPN can be further categorized into diabetic sensory polyneuropathy and autonomic neuropathy. It shows the earliest onset and is the most prevalent diabetic micorangiopathy [1]. DPN is a sensory-dominant neuropathy and symptoms include hyperalgesia and/or hypoalgesia, pain, allodynia, paralysis, and ulcer formation. Pain can lead to insomnia as well as incurable ulcerations and infections that may eventually lead to lower-limb amputations, which reduce patient quality of the life. Furthermore, indolent acute myocardial infarction and arrhythmia due to autonomic neuropathy can dramatically worsen prognosis [3]. Intraepidermal small nerve fibers of the foot skin can decrease even if subjective symptoms are not evident in the early stages of DPN [4]. This reduction of small nerve fibers is progressive in DPN and can subsequently or simultaneously develop into degeneration of large myelinated fiber and demyelination. At present, there is no established radical treatment for DPN, and lifestyle intervention and strict glycemic control play significant roles in preventing DPN progression.

The current understanding of the mechanisms involved in DPN is based on aberrant glucose metabolism [1,5]. The incidence of metabolic syndrome modifies the pathology of DPN in type 2 diabetes [6,7]. Hyperglycemia can activate multiple collateral glucose-utilizing pathways, such as the polyol pathway, protein kinase C (PKC) pathway, advanced glycation end-products (AGEs) formation, hexosamine biosynthetic pathway, pentose phosphate pathway, and anaerobic glycolytic pathway [1,5,8,9,10]. These mechanisms are assumed to individually or synergistically trigger the onset and progression of DPN. We previously reported that these mechanisms may sequentially contribute to the pathophysiology of small fiber neuropathy related to abnormal glucose metabolism [11,12,13]. Initially, low levels of oxidative stress are involved in the pathogenesis of small fiber neuropathy in patients with normal-high HbA1c levels [11,13]. Thereafter, oxidative stress level gradually increases in proportion to DPN stage [12,13]. The implication of inflammation is evident from impaired fasting glucose levels [12,13], and AGEs are eventually observed at the advanced stages of diabetes [12]. Similarly, hyperactivation of the polyol pathway in the peripheral nerves of DPN is implicated in the initial stages of DPN [14,15]. These findings suggest that glucose metabolism may be altered in the peripheral nerves depending on DPN stage and state. It is important to understand these changes to establish a suitable therapy for DPN. In the present review, recent developments into the mechanisms involved in DPN are introduced with a focus on glucose metabolism, particularly via the collateral glucose-utilizing pathways, in the peripheral nerves in DPN.

## 2. Glucose Metabolism in the Peripheral Nerves

The pathogenesis of diabetes involves insulin resistance in the peripheral tissues, such as adipose tissue, muscle, and liver, and a lack of insulin secretion from pancreatic β-cells, leading to abnormal glucose metabolism. Similarly, abnormal glucose metabolism is elicited by insulin resistance in the peripheral nerves in DPN, and is common between type 1 and type 2 diabetes [16]. Nevertheless, there are clear differences in the pathogenesis of DPN between type 1 and type 2 diabetes [2,17,18]. In type 1 diabetes, strict blood glucose control is more effective to suppress the development of DPN, whereas it is difficult to deplete the progression of DPN in type 2 diabetes only using glycemic control due to the contribution of factors related to metabolic syndrome, such as dyslipidemia and obesity [2,17,18].

**Figure 1:**
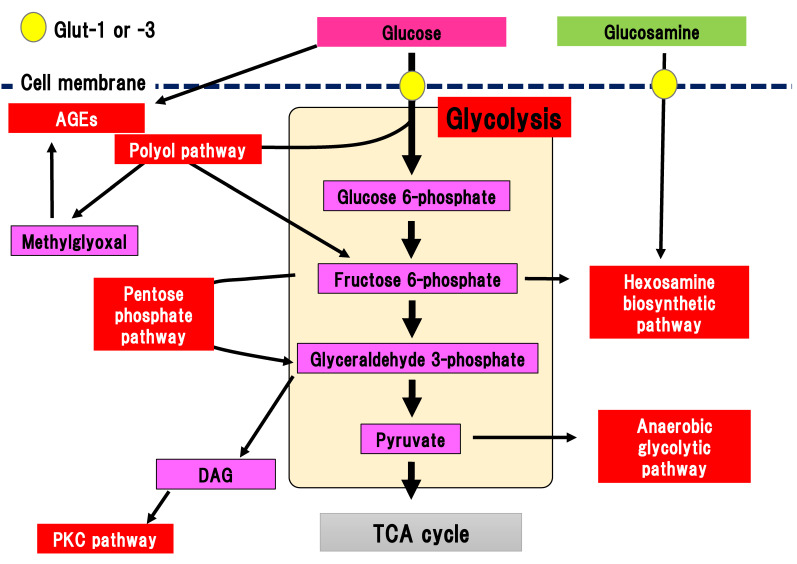
Collateral glucose-utilizing pathways associated with diabetic polyneuropathy. Glycolysis is a central pathway involved in glucose metabolism. There are several collateral glucose-utilizing pathways, such as the polyol pathway, PKC pathway, glycation, hexosamine biosynthetic pathway including glucosamine metabolism, pentose phosphate pathway, and anaerobic glycolytic pathway. AGE, advanced glycation end-product; DAG, diacylglycerol; Glut, glucose transporter; PKC, protein kinase C; TCA cycle, tricarboxylic acid cycle.

Glucose is transported into the cytosol by the glucose transporters, Glut-1 and Glut-3, in the peripheral nerves (Figure 1) [19,20].

Glucose transportation via Glut-1 and Glut-3 is independent on the effects of insulin, and differs from the effects of Glut-4 expressed in adipose tissue. Transported glucose is metabolized via glycolysis. Glycolysis is a fundamental glucose metabolic pathway in which glucose is step-wisely phosphorylated and metabolized in cells. Almost all the reactions in glycolysis are reversible, although the reactions catalyzed by hexokinase, phosphofructokinase, and pyruvic acid kinase are irreversible. Pyruvic acid (pyruvate), which is the final metabolite of glycolysis, is transported into the TCA cycle in the mitochondria under the aerobic conditions. Pyruvate is metabolized into lactic acid and released from the cells in anaerobic conditions. Glut-4 dysfunction is evident in the adipose tissue of patients with diabetes, resulting in reduced incorporation of glucose. In contrast, dysfunction of Glut-1 and Glut-3 is not apparent in the peripheral nerves in DPN. Our previous comprehensive metabolome analysis revealed that sequential metabolites involved in glycolysis are presrved in the sciatic nerve tissues of streptozotocin (STZ)-induced type 1 diabetic mice [15]. Therefore, changes to Glut expression and their dysfunction may minimally contribute to the pathogenesis of DPN, even in the peripheral nerves in DPN.

There are several collateral pathways associated with glycolysis, such as the polyol pathway, hexosamine biosynthetic pathway including glucosamine metabolism, PKC pathway, AGEs pathway, pentose phosphate pathway, and anaerobic glycolytic pathway (Figure 1). In the peripheral nerves, collateral pathways are minimally activated in the non-diabetic state. In contrast, since glucose uptake into the cytosol and flux into the glycolytic pathway are increased in diabetes, collateral pathways associated with glycolysis are simultaneously activated. For example, glucose flux into the polyol pathway is increased three to four-fold compared with the non-diabetic state. Activated collateral pathways are known to be differently implicated in the pathogenesis of DPN.

## 3. Collateral Pathways of Glycolysis and the Pathogenesis of DPN

### 3.1. Polyol Pathway

**Figure 2:**
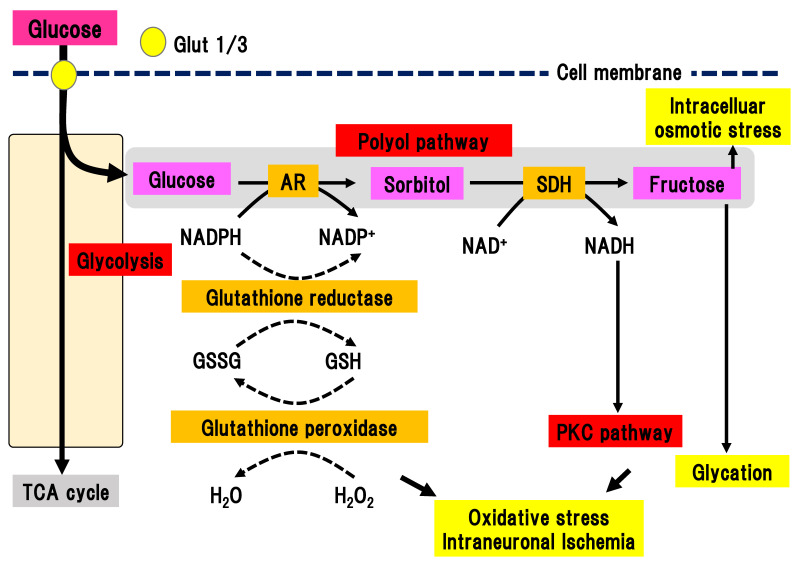
Polyol pathway. Excess glucose is metabolized by the polyol pathway into sorbitol in a reaction catalyzed by aldose reductase. Activation of this pathway elicits oxidative stress, intraneuronal ischemia, and increased intracellular osmotic stress. This pathway also connects with the PKC pathway and glycation. AR, aldose reductase; Glut, glucose transporter; GSH, glutathione; GSSG, glutathione disulfide; NAD(H), nicotinamide adenine dinucleotide; NADP(H), nicotinamide adenine dinucleotide phosphate; PKC, protein kinase C; SDH, sorbitol dehydrogenase; TCA cycle, tricarboxylic acid cycle.

In the polyol pathway, glucose is metabolized to sorbitol in a reaction catalyzed by aldose reductase, which is a rate-limiting enzyme (Figure 2) [21,22].

In the polyol pathway, nicotinamide adenine diphosphate (NADPH) is simultaneously converted to NADP^+^. Activation of aldose reductase consumes NADPH, resulting in a reduced ability to defend against oxidative stress. Subsequently, sorbitol dehydrogenase metabolizes sorbitol into fructose, leading to the conversion of NAD to NADH. This reaction leads to an imbalance of the osmotic pressure in the cytosol, osmotic stress, and subsequent efflux of myoinositole and taurine. The shortage of myoinositole is directly linked with low activation of Na^+^/K^+^ ATPase and a decline in cell function. Myoinositol insufficiency can also elicit the production of diacylglycerol (DAG), which is an activator of PKC. Aldose reductase inhibitors (ARIs) are the only clinically approved compound used as a therapeutic agent for DPN in Japan. Hotta et al. revealed that the application of ARIs significantly ameliorated the delay of nerve conduction velocity in Japanese DPN patients, although its effects were shown to be limited in patients with poor glycemic control [14]. This finding suggests that glucose may shift to another collateral pathway in glycolysis due to inhibition of the polyol pathway by ARIs. On the other hand, ARIs had a marginal effect on the Caucasian subjects with DPN in most double-blinded studies [23], implying differences in the pathophysiology of DPN between Caucasian and Japanese patients. There may be factors associated with metabolic syndrome that are more dominant in Caucasian patients with diabetes compared with Japanese patients.

### 3.2. PKC Pathway

**Figure 3:**
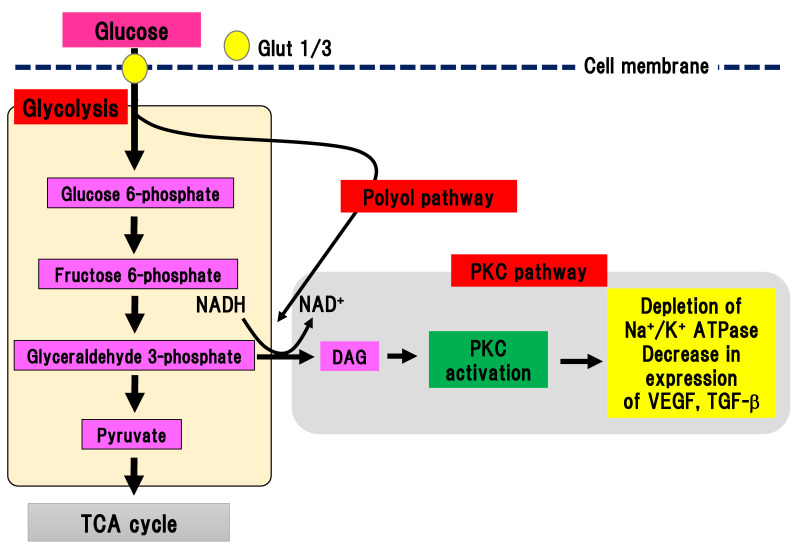
PKC pathway. Glyceraldehyde 3-phosphate is converted to phosphatidic acid, resulting in the accumulation of DAG, which activates PKC. On the other hand, the polyol pathway can activate the PKC pathway by increasing the production of NADH. Glut, glucose transporter; DAG, diacylglycerol; NAD(H), nicotinamide adenine dinucleotide; PKC, protein kinase C; TCA cycle, tricarboxylic acid cycle; TGF-β, transforming growth factor-β; VEGF, vascular endothelial growth factor.

PKC is a serine/threonine kinase involved in various cell signaling pathways [24]. Activation of PKC leads to cell differentiation, proliferation, and death. Continuous hyperglycemia increases the generation of glyceraldehyde 3-phosphate, an intermediate product of glycolysis, which is converted to phosphatidic acid, resulting in the accumulation of DAG (Figure 3).

DAG accumulation can activate PKC. Interestingly, PKC activation in DPN is different depending on the organ involved. In endothelial cells and smooth muscle cells in blood vessels, PKC can be activated, while its activity is decreased in neuronal cells and Schwann cells [25,26]. Activation of PKC reduces Na^+^/K^+^ ATPase activity, expression of vascular endothelial growth factor and TGF-β, and ischemia and hypoxia in tissues. In an experimental model of DPN, inhibition of PKC-β ameliorated hyperalgesia in DPN [27]. However, since no beneficial effects of inhibition of human DPN were found, clinical application has not been achieved.

### 3.3. AGEs Pathway

**Figure 4:**
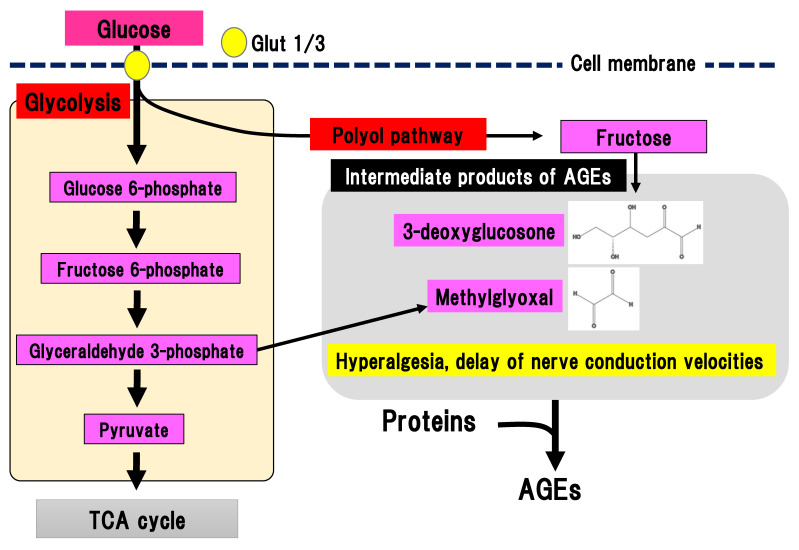
AGEs pathway. Fructose, which is an end-product of the polyol pathway, is converted to 3-deoxyglucosone and glyceraldehyde 3-phosphate is converted to methylglyoxal. 3-deoxyglucosone and methylglyoxal are intermediate products of AGEs. AGEs, advanced glycation end-products: Glut, glucose transporter; TCA cycle, tricarboxylic acid cycle.

During chronic hyperglycemia, the aldehyde base in reducing sugars such as glucose reacts with the amino group in proteins in a non-enzymatic manner, resulting in the generation of nitrogen glucoside, Schiff base, Amadori compound, and AGEs. Intermediate products of AGEs are also generated from glycolysis (Figure 4).

Binding of AGEs to their receptor in macrophages leads to nuclear translocation of NF-kB and an inflammatory reaction, and tissue injury [28]. AGEs may contribute to the pathogenesis of DPN since the concentration of N(ε)-carboxymethyllysine, a type of AGEs, is increased in the sciatic nerves of STZ-induced diabetes animal models [29]. Glycolysis generates dicarbonyl compounds such as glyoxal, methylglyoxal, and 3-deoxyglucosone. Glyceraldehyde 3-phosophate is converted to dihydroxyacetone phosphate, catalyzed by triosephosphate isomerase, and methylglyoxal, catalyzed by methylglyoxal synthase. Fructose is an end-product of the polyol pathway and is further converted to fructose 3-phospate and 3-deoxyglucosone. Although dicarbonyl compounds are intermediate products of AGEs, they have a higher reactivity than AGEs. Particularly, methylglyoxal induces the activation of nociceptive receptors via the voltage-gated sodium channel, Na(v)1.8, resulting in hyperalgesia and delayed nerve conduction velocity in mice [30]. Nav1.8 knockout in mice prevented the effects of methylglyoxal on the peripheral nerves in experimental DPN. Therefore, suppression of methylglyoxal generation is expected to be a promising treatment for painful DPN. On the other hand, excessive activation of Nav1.8 may be prevented by suppression of glyceraldehyde 3-phoshphate production in glycolysis.

### 3.4. Hexosamine Biosynthetic Pathway

**Figure 5:**
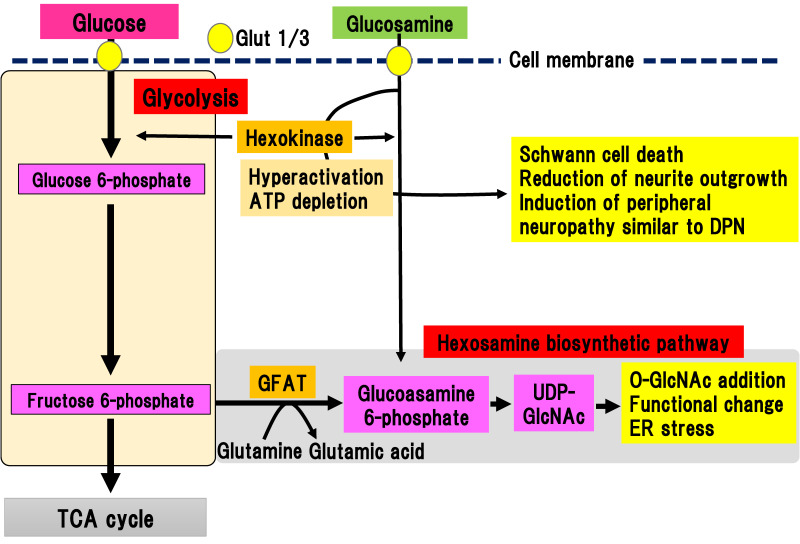
Hexosamine biosynthetic pathway and glucosamine pathway. Fructose 6-phosphate is converted to glucosamine 6-phosphate by glutamine-fructose-6-phosphate aminotransferase (GFAT). Glucosamine 6-phosphate is converted to uridine diphosphate-*N*-acetylglucosamine (UDP-GlcNAc), which can bind to serine/threonine residues in proteins via O-linked-glycosylation. Extracellular glucosamine is transported into the cytosol by Glut 1 or Glut 3. Incorporated glucosamine is initially metabolized to glucosamine 6-phosphate by hexokinase, consuming ATP. Glucosamine 6-phosphate is used in the hexosamine biosynthetic pathway. ER, endoplasmic reticulum; GFAT, glutamine-fructose-6-phosphate aminotransferase; Glut, glucose transporter; TCA cycle, tricarboxylic acid cycle; UDP-GlcNAc, uridine diphosphate (UDP)-*N*-acetylglucosamine.

The hexosamine biosynthetic pathway is responsible for 2% to 3% of glucose metabolism in glycolysis [31]. Since glucose flux is increased in hyperglycemia, the hexosamine biosynthetic pathway is also activated in diabetes [32]. The hexosamine biosynthetic pathway stems from glycolysis and begins with a reaction that converts fructose 6-phosphate to glucosamine 6-phosphate, catalyzed by glutamine-fructose-6-phosphate aminotransferase (GFAT) (Figure 5).

The final metabolite of this pathway is uridine diphosphate (UDP)-*N*-acetylglucosamine (UDP-GlcNAc), which can bind to serine or threonine residues in proteins via *O*-linked-glycosylation, resulting in post-translational modifications by monomeric *O*-linked *N*-acetyl-d-glucosamine (O-GlcNAc). The hexosamine biosynthetic pathway has been suggested to function together with O-GlcNAc modification of proteins as a nutrient sensor in cells [33,34]. This reversible glycosylation can modulate the activity of many types of protein, such as the phosphorylation of kinases and transcriptional activity of transcriptional factors [35]. The hexosamine biosynthetic pathway is also activated by the metabolism of glucosamine into glucosamine 6-phosphate, which is directly incorporated via glucose transporters into the extracellular matrix. Increased expression of GFAT in pancreatic β-cells led to generation of hydrogen peroxide and decreased expression of insulin, Glut-2, and glucokinase, resulting in β-cell dysfunction [36]. In complications of diabetes, the hexosamine biosynthetic pathway is implicated in the upregulation of TGF-β expression and increase in mesangeal matrix [37]. This is ascribed to the activation of the transcriptional factor, sp1, due to the activation of the hexosamine biosynthetic pathway, which triggers endoplasmic reticulum (ER) stress [38]. Similarly, ER stress is involved in the pathogenesis of DPN [39]. Although, there is indirect evidence for the implication of the hexosamine biosynthetic pathway in the pathogenesis of DPN, no direct evidence has been shown.

### 3.5. Glucosamine Pathway

Glucosamine is a precursor of UDP-GlcNAc, the main product of the hexosamine biosynthetic pathway, and is often used to mimic its activation (Figure 5). In addition to activation of the hexosamine biosynthetic pathway, Glucosamine flux competitively inhibits glucose uptake, downregulating glucokinase expression in pancreatic β-cells or hepatocytes, resulting in induction of ER stress or apoptotic cell death independent of the hexosamine biosynthetic pathway [36]. Lim et al. showed that glucosamine acutely reduces cellular glucose uptake, glucokinase activity, and intracellular ATP levels in induced motor neuronal cells [40]. As a result, AMP-activated protein kinase activity and ER stress increased, leading to a reduction of cell viability [40]. Although these results suggest a neurotoxic role of glucosamine, even in the peripheral nerves, detailed studies have not been conducted due to a lack of information concerning the amount of glucosamine in nerve tissues.

We recently reported the detailed roles of glucosamine in experimental DPN models of type 1 diabetes [15]. We evaluated the amount of glucosamine accumulation in the sciatic nerve of STZ-induced type 1 diabetic mice [15]. Comprehensive metabolomic analysis revealed a marked accumulation of glucosamine in the sciatic nerves, regardless of the presence of aldose reductase gene. In vitro analysis showed that glucosamine stimulation induced cell death of a Schwann cell line and inhibition of neurite outgrowth in primary cultured dorsal root ganglia (DRG) neuronal cells. Glucosamine was shown to excessively activate glucokinase, which is a rate-limiting enzyme of glucosamine metabolism, resulting in the depletion of ATP. Short and long-term administration of glucosamine into non-diabetic mice resulted in sensory-dominant neuropathy similar to DPN. These results suggest that glucosamine contributes to the onset and progression of experimental DPN. Concurrently, our results may imply that the hexosamine biosynthetic pathway may partially contribute to the development and onset of DPN, as well.

### 3.6. Pentose Phosphate Pathway

**Figure 6:**
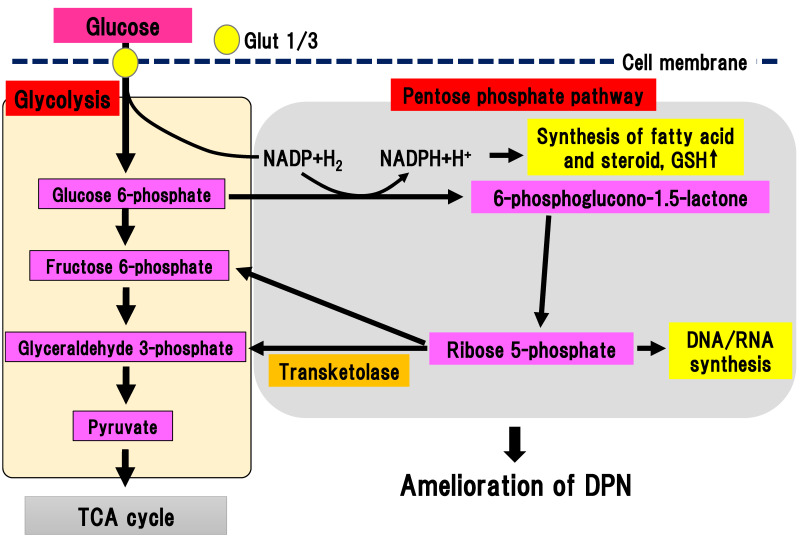
Pentose phosphate pathway. Glucose 6-phosphate is metabolized to a series of pentoses, such as ribose 5-phosphate. Transketolase catalyzes the conversion of ribose 5-phosphate into fructose 6-phosphate and glyceraldehyde 3-phosphate. DPN, diabetic polyneuropathy; Glut, glucose transporter; GSH, glutathione; NADP(H), nicotinamide adenine dinucleotide phosphate; TCA cycle, tricarboxylic acid cycle.

In the pentose phosphate pathway, glucose 6-phosphate is metabolized to a series of pentoses, such as ribose 5-phosphate (Figure 6).

As final metabolites in pentose phosphate pathway, fructose 6-phosphate and glyceraldehyde 3-phosphate are produced, generating NADPH, which is an antioxidant substance necessary for lipid synthesis. Ribose 5-phosphate is a source of nucleic acid. One cycle of the pentose phosphate pathway generates one molecule of carbon dioxide and two molecules of NADPH from one molecule of glucose 6-phophate. Transketolase depends on thiamine 2-phosphate, which can catalyze several important steps in the pentose phosphate pathway. In a physiological state, transketolase is activated depending on the concentration of the substrate [41]. When glucose 6-phosphate and fructose 6-phosphate are saturated in a hyperglycemic state, transketolase can be activated and reused to produce pentose 5-phosphate and erythrose 4-phosphate. In experimental diabetic retinopathy, symptoms were ameliorated by activation of transketolase [42]. In new onset DPN within one year, the single nucleotide polymorphism (SNP) rs7648309 in transketolase was significantly correlated with an elevated total symptom score, and the rs62255988 SNP was correlated with delayed thermal threshold [8]. In an experimental DPN model, activation of glucose 6-phosphate dehydrogenase facilitated flux into the pentose phosphate pathway, resulting in inhibition of neuronal death due to hyperglycemia [9]. Thus, activation of the pentose phosphate pathway could ameliorate DPN. Benfotiamine (S-benzoylthiamine O-monophosphate) is an antioxidant compound that can ameliorate DPN symptoms. It has anti-DPN effects with multiple sites of action, including the activation of transketolase in the pentose phosphate pathway [42]. The efficacy and safety of benfotiamine has been investigated in patients with DPN in several randomized, double-blind clinical trials for 3–12 weeks [43,44,45,46]. All trials using benfotiamine showed significant improvement of neuropathic symptoms in DPN patients [43,44,45,46]. Nevertheless, there is a paucity of promising data regarding the long-term use of benfotiamine treatment for human DPN. This suggests that the effects of monotherapy may be less remarkable during the course of DPN since other pathways may be activated due to the multifactorial pathophysiology of DPN.

### 3.7. Anaerobic Glycolytic Pathway

**Figure 7:**
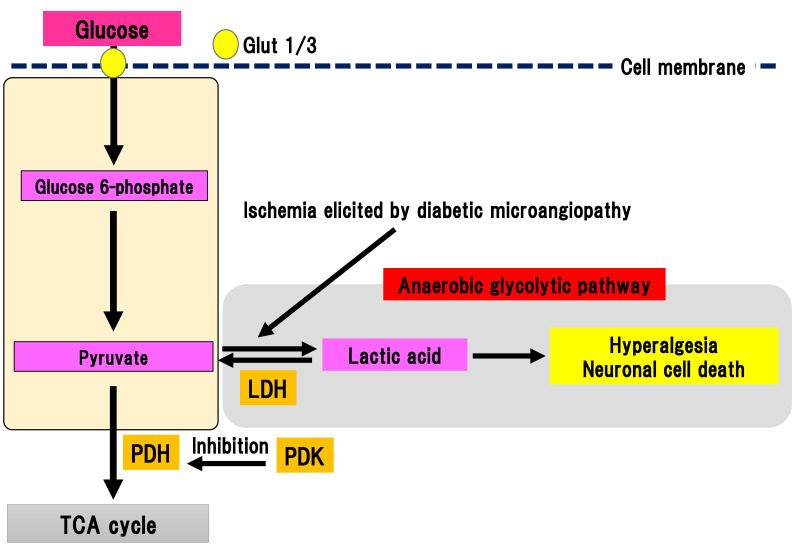
Anaerobic glycolytic pathway. Pyruvate is metabolized to lactic acid by lactate dehydrogenase in anaerobic conditions evoked by ischemia in diabetic microangiopathy. Glut, glucose transporter; LDH, lactate dehydrogenase; PDH, pyruvate dehydrogenase; PDK, PDH kinase; TCA cycle, tricarboxylic acid cycle.

Pyruvate is the final metabolite in the aerobic glycolytic pathway (Figure 7).

Pyruvate is transferred to the TCA cycle and electron transport chain reaction in mitochondria, resulting in the generation of 32 ATP molecules as a final product. Conversely, pyruvate can be metabolized to lactic acid by lactate dehydrogenase in anaerobic conditions. Anaerobic glycolytic pathway generates two ATP molecules and one lactate molecule from one glucose molecule. In DPN, peripheral nerves are under chronic ischemia because of the thickness and narrowing of microvessels due to diabetic microangiopathy, which lowers the intranervous blood flow [47]. Thus, anaerobic glycolytic pathway tends to be activated in the peripheral nerves with DPN. Pyruvate dehydrogenase (PDH) is a rate-limiting enzyme in TCA cycle [48]. It can oxidize carboxyl groups resulting in the production of acetyl-CoA, NADH, and carbon dioxide. PDH is phosphorylated by PDH kinase (PDK), which can suppress the activation of PDH. There are four subtypes of PDK: PDK1-4 [49]. PDK is widely expressed in human tissues [50]. Specific PDK subtypes are activated in specific situations (e.g., PDK1 in anaerobic conditions, PDK2 in high acetyl-CoA and NADH conditions, PDK3 in high ATP conditions, and PDK4 in starvation and a state of low nutrition) [51]. Activation of PDK subsequently suppresses activation of the TCA cycle, resulting in activation of anaerobic glycolysis and an increase in lactic acid production. The four different PDK isoforms are expressed in diverse peripheral and central tissues [52]. Activation of PDK in the peripheral nerves of DPN was reported [10]. In an STZ-induced diabetes model, expression of PDK2 and PDK4 were increased with activation in DRG. Double knockout of PDK2 and PDK4 ameliorated hyperalgesia, an activation of ion channels associated with pain sensation and satellite glial cells, and macrophage infiltration accompanied by suppression of increase in lactate. In primary culture of DRG cells, the addition of lactate to the medium increased the expression of ion channels and decrease cell viability. Thus, these results suggest that anaerobic metabolism is implicated in the pathophysiology of DPN in an STZ model. Anaerobic metabolism can decrease the viability of DRG neurons and trigger hyperalgesia. Furthermore, dichloroacetic acid, a PDK inhibitor, or FX11, a lactate dehydrogenase inhibitor, could partially alleviate hyperalgesia in an STZ model. These results indicate that PDK may be a target for painful diabetic neuropathy treatment.

## 4. Future Perspective for Therapeutic Application

The pathophysiology of DPN changes during its course [11,12,13]. Because of these changes, the efficacy of ARI can be limited and benfotiamine shows no long-term efficacy in a real-world clinical setting of DPN. These suggest that each collateral glucose-utilizing pathway may play a partial role in the certain periods of DPN development. In other words, each pathway can be activated in the different stages or situations of diabetes and DPN. Furthermore, it is also possible that synergistic effects of the concurrent activation of these pathways may be ascribed to DPN development. In addition, from the view that the pathogenesis of DPN is multifactorial, it is important to consider the contribution of factors other than glucose metabolism to the pathogenesis of DPN. The factors of metabolic syndrome such as obesity, dyslipidemia, and hypertension are known to be risk for DPN, which may trigger inflammatory reactions [6,12]. These factors can also influence the activity of each collateral glucose-utilizing pathways. Therefore, it is important to know the whole changes of the metabolic pathway in the peripheral nerve tissues in human DPN to introduce the therapies to which the collateral glucose-utilizing pathways are applied. To achieve this end, comprehensive metabolomic analyses should be performed in the peripheral nerves of human DPN, which would improve our understanding of the whole changes in the metabolism including glucose flux into the collateral pathways. Such comprehensive analyses may give us the information how to apply the therapies, and further elucidate the hidden changes in glucose metabolism in DPN, such as glucosamine accumulation [15].

## 5. Conclusions

Various collateral glucose-utilizing pathways of glycolysis can be differently or synergistically activated in response to increases in glucose flux in the peripheral nerves of DPN, which can contribute to the pathophysiology of DPN. It is crucial to identify the timings of activation and selections of those pathways for the therapeutic application. In order to establish the radical treatment of DPN including the application of collateral glucose-utilizing pathways, we should also consider the whole changes of metabolism including other factors than glucose in the peripheral nerves of DPN. Therefore, it is expected to identify compounds that have multiple and heterochronous targets and comprehensively treat DPN patients.

## Abbreviations

DPN	Diabetic polyneuropathy
PKC	Protein kinase C
AGEs	Advanced glycation end-products
Glut	Glucose transporter
DAG	Diacylglycerol
TCA	Tricarboxylic acid
STZ	Streptozotocin
AR	Aldose reductase
NADP(H)	Nicotinamide adenine dinucleotide phosphate
GSSG	Glutathione disulfide
GSH	Glutathione
SDH	Sorbitol dehydrogenase
NAD(H)	Nicotinamide adenine dinucleotide
ARI	Aldose reductase inhibitor
VEGF	Vascular endothelial growth factor
TGF-β	Transforming growth factor-β
GFAT	Glutamine-fructose-6-phosphate aminotransferase
UDP-GlcNAc	Uridine diphosphate (UDP)-N-acetylglucosamine
O-GlcNAc	O-linked N-acetyl-d-glucosamine
DRG	Dorsal root ganglia
ER stress	Endoplasmic reticulum stress
SNPs	Single nucleotide polymorphisms
Benfotiamine	S-benzoylthiamine O-monophosphate
LDH	Lactate dehydrogenase
PDH	Pyruvate dehydrogenase
PDK	PDH kinase
